# Retinal Amino Acid Neurochemistry of the Southern Hemisphere Lamprey, *Geotria australis*

**DOI:** 10.1371/journal.pone.0058406

**Published:** 2013-03-13

**Authors:** Lisa Nivison-Smith, Shaun P. Collin, Yuan Zhu, Sarah Ready, Monica L. Acosta, David M. Hunt, Ian C. Potter, Michael Kalloniatis

**Affiliations:** 1 School of Optometry and Vision Science, University of New South Wales, Sydney, New South Wales, Australia; 2 School of Animal Biology and the University of Western Australia Oceans Institute, University of Western Australia, Crawley, Western Australia, Australia; 3 School of Biomedical Sciences, The University of Queensland, Brisbane, Queensland, Australia; 4 Queensland Brain Institute, University of Queensland, Brisbane, Queensland, Australia; 5 Department of Optometry and Vision Science, University of Auckland, Auckland, New Zealand; 6 School of Biological Sciences and Biotechnology, Murdoch University, Murdoch, Western Australia, Australia; 7 Centre for Eye Health, University of New South Wales, Sydney, New South Wales, Australia; Dalhousie University, Canada

## Abstract

Lampreys are one of the two surviving groups of the agnathan (jawless) stages in vertebrate evolution and are thus ideal candidates for elucidating the evolution of visual systems. This study investigated the retinal amino acid neurochemistry of the southern hemisphere lamprey *Geotria australis* during the downstream migration of the young, recently-metamorphosed juveniles to the sea and during the upstream migration of the fully-grown and sexually-maturing adults to their spawning areas. Glutamate and taurine were distributed throughout the retina, whilst GABA and glycine were confined to neurons of the inner retina matching patterns seen in most other vertebrates. Glutamine and aspartate immunoreactivity was closely matched to Müller cell morphology. Between the migratory phases, few differences were observed in the distribution of major neurotransmitters i.e. glutamate, GABA and glycine, but changes in amino acids associated with retinal metabolism i.e. glutamine and aspartate, were evident. Taurine immunoreactivity was mostly conserved between migrant stages, consistent with its role in primary cell functions such as osmoregulation. Further investigation of glutamate signalling using the probe agmatine (AGB) to map cation channel permeability revealed entry of AGB into photoreceptors and horizontal cells followed by accumulation in inner retinal neurons. Similarities in AGB profiles between upstream and downstream migrant of *G. australis* confirmed the conservation of glutamate neurotransmission. Finally, calcium binding proteins, calbindin and calretinin were localized to the inner retina whilst recoverin was localized to photoreceptors. Overall, conservation of major amino acid neurotransmitters and calcium-associated proteins in the lamprey retina confirms these elements as essential features of the vertebrate visual system. On the other hand, metabolic elements of the retina such as neurotransmitter precursor amino acids and Müller cells are more sensitive to environmental changes associated with migration.

## Introduction

**Figure 1:**
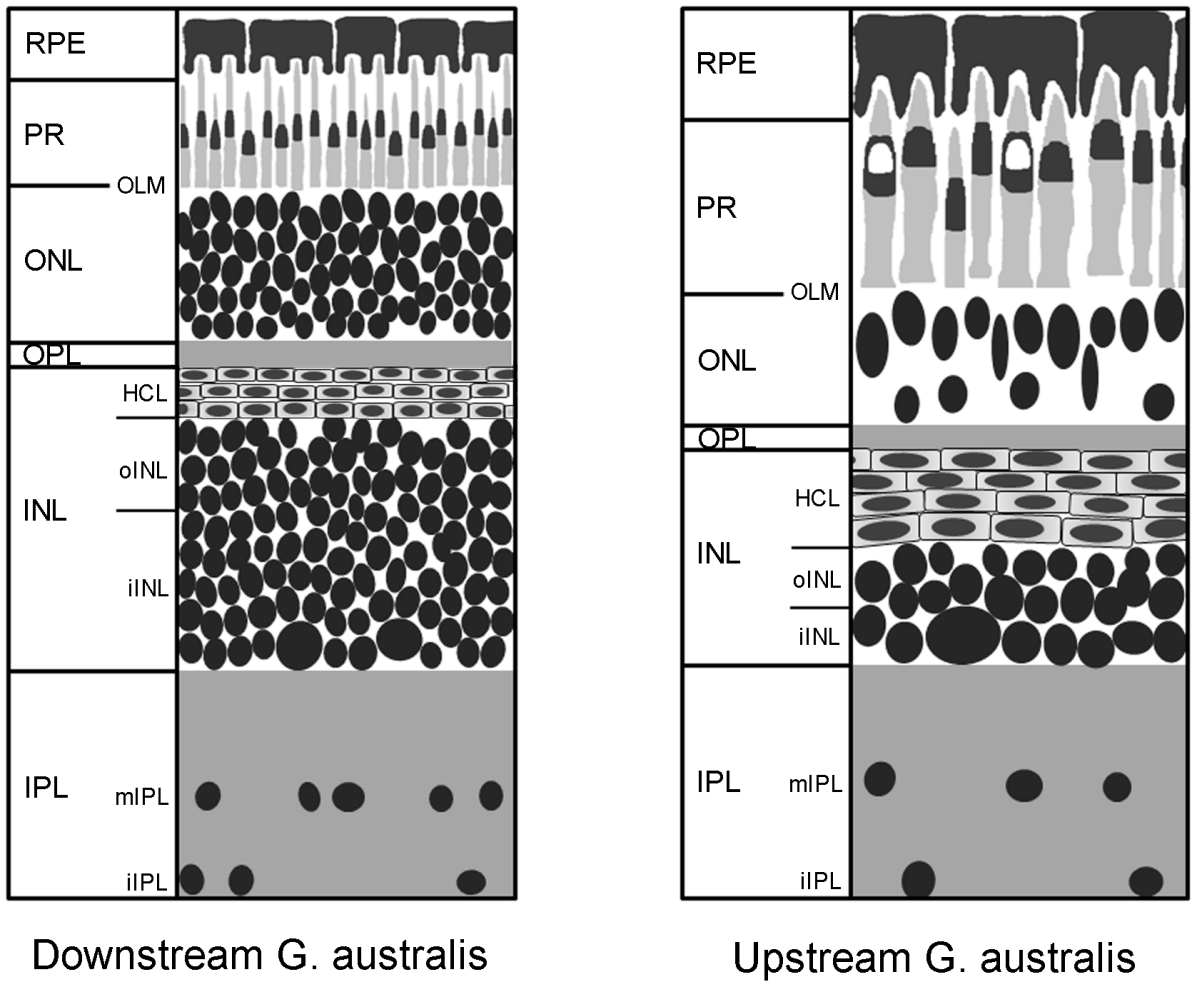
Schematic of the retina of downstream and upstream migrants of G australis. The downstream *G. australis* retina is densely packed with over 10 layers of cells in the INL. The upstream *G. australis* retina is much larger; dominated by large photoreceptors and a reduced number of cell layers in the INL. The majority of the ganglion cells are displaced to the INL and a few orthotopic ganglion cells lie at the vitread border of the IPL and in the middle of the IPL. Figure is not drawn to scale. Abbreviations - RPE: retinal pigment epithelium, PR: photoreceptors, OLM: outer limiting membrane, ONL: outer nuclear layer, OPL: outer plexiform layer, INL: inner nuclear layer, HCL: horizontal cell layer, oINL: outer INL, iINL: inner INL, IPL: inner plexiform layer, mIPL: mid IPL, iIPL: inner IPL.

Lampreys, together with the hagfishes, are the sole survivors of the early agnathan (jawless) stages in vertebrate evolution [1], [2]. The fully differentiated lamprey eye possesses many similar features to the human eye including extra-ocular muscles, a multifocal lens and optic nerve [3]–[7]. The lamprey retina conforms to the common structure and composition observed in most vertebrate eyes [8]. Neurons are organised into discrete layers of cell bodies and axonal and dendritic processes; i.e. nuclear and plexiform layers. Photoreceptors are located sclerad to second and third order neurons, including bipolar, horizontal, amacrine and ganglion cells situated closer to the vitreous [9]–[11]. Interestingly, the ganglion cells in the lamprey retina are mostly displaced to the inner nuclear layer (INL) near the inner plexiform layer (IPL) border with few ganglion cells situated in the middle of the IPL and adjoining the inner limiting membrane (Figure 1; [12], [13]).

Beyond anatomical similarities, the expression of key phototransduction proteins including transducin, phosphodiesterase 6 and recoverin in the lamprey retina indicates the presence of a characteristic vertebrate visual system [14]–[17]. Immunoreactivity of retinal cell markers like calbindin (CalB), calretinin (CalR), choline acetyltransferase (ChAT) and glial fibrillary acidic protein (GFAP) have also been shown in the lamprey inner retina [16], [18]–[21]. Characterisation of neurochemical pathways in the lamprey is limited. The major retinal amino acid neurotransmitters, glutamate, γ-aminobutyric acid (GABA) and glycine are present in the adult lamprey retina and show similar distribution patterns to other vertebrate retinae [22]. Other molecules such as dopamine and serotonin have also been detected although their role in the lamprey retina is unclear [23], [24]. Overall, current data suggests that the lamprey can provide clues to the evolution of the visual and nervous systems of gnathostome vertebrates.

The majority of lamprey species are found in the northern hemisphere and belong to the family Petromyzontidae [25]. Extensive work has focused on northern hemisphere species, particularly *Petromyzon marinus*, *Lampetra fluviatilis* and *Ichthyomyzon unicuspis.* These animals have two structurally and spectrally distinct photoreceptor types, proposed as a rod-like and a cone-like photoreceptor although this classification is still subject to debate (reviewed in [3], [26], [27]). Four species of lamprey are native to the southern hemisphere and have been placed into two families, the Geotriidae and Mordaciidae [28], [29]. *Geotria australis* is the only member of the Geotriidae and is found in New Zealand, Southern Australia, Chile and Argentina [28], [29]. Unlike its northern hemisphere relatives, the retina of *G. australis* contains five distinct cone-like photoreceptors and five different opsin proteins [3], [30]–[32]. These opsins have been identified as direct orthologues of the long wavelength sensitive (LWS), short wavelength sensitive (SWS1 and SWS2) and medium wavelength sensitive (RHA/RH1 and RHB/RH2) opsins found in jawed vertebrates [31], [33], [34].

Here, we seek to further understand the neurochemistry of the lamprey retina by investigating the amino acid distribution in the retina of *G. australis*. *G. australis* was selected as it features a complex (possibly pentachromatic) visual system, which may provide greater insight into the neurochemistry of colour vision than the dichromatic visual system of its northern hemisphere counterparts [35]. Furthermore, the retinal plasticity observed between the different migratory phases of *G. australis* (Figure 1) provides a unique opportunity to investigate changes in neurochemical pathways according to environmental pressures. Downstream migrants of *G. australis* (characterized as recently metamorphosed adults travelling from freshwater rivers to the ocean) possess a short and densely packed photoreceptor layer, likely to reflect its highly photopic habitat of shallow fresh water rivers [30], [32], [36]. However, upstream migrants (older fish returning from the sea into freshwater to breed) have retinas dominated by large photoreceptor cells [30], [31], [37]. The yellow myeloid pigment present in one of the photoreceptor types (C2) of the downstream migrants is also replaced in the upstream migrating *G. australis* with a large unpigmented ellipsosome, resulting in a shift in the peak spectral sensitivity (λ_max_) from 492 nm to 552 nm [30], [32], [38]. These changes possibly reflect selective pressures produced by the changing light environment as adult lampreys in the ocean avoid avian predators at the surface when they move back up to their natal streams by adopting a scotopic lifestyle [3], [30]. In this study, we will investigate key amino acid neurotransmitters in the retina of *G. australis* using immunocytochemical techniques and determine if their distribution is associated with the anatomical differences found in the retina of downstream and upstream migrants. We will also investigate glutamate signalling by analysing cation channel permeability using the small organic cation, agmatine (AGB) and briefly assess the role of calcium, based on the distribution of calcium binding proteins.

## Materials and Methods

### Ethics Statement

This study was carried out in strict accordance with the recommendations in the Guide for the Care and Use of Laboratory Animals of the National Institutes of Health. Animals were killed with an overdose of methane tricaine sulfonate salt in concordance with ethical guidelines of the National Health and Medical Research Council of Australia, and all efforts were made to minimize suffering. All experimental protocols were approved by The University of Queensland Animal Ethics Committee.

### Animals

Adult *G. australis* were collected as young, downstream migrants (75–110 mm in length; n = 9) or upstream migrants (560–640 mm in length; n = 8) from streams and rivers in south-western Australia using an electric fish shocker as described previously [3]. Animals were maintained in laboratory aquaria at 17°C under a 12 h light/dark cycle under Queensland Government Department of Primary Industries General Fisheries Permit, Stock Impoundment No: PRM01814G.

### Post-embedding Immunocytochemistry

The procedures for post-embedding immunocytochemistry have been previously described [39]–[42]. Briefly, eyes were enucleated and isolated retinal pieces were immediately fixed in 2.5% (w/v) glutaraldehyde, 1% (w/v) paraformaldehyde in 0.1 M phosphate buffer (PB, pH 7.4) for at least 30 min to preserve amino acid neurochemistry. Retinal pieces were washed in PB and dehydrated through a series of cold methanol washes to acetone followed by impregnating in resin. Resin blocks were serially sectioned at 0.25 µm and collected on Teflon-coated slides. Before incubation with primary antibodies, the resin was etched with sodium ethoxide:ethanol (1∶5) and the tissue washed with a graded methanol series to PB. Tissues were then incubated with 1% (w/v) sodium borohydride for 30 min and air dried.

Primary antibodies for glutamate, glycine, glutamine, GABA, taurine and aspartate were diluted with 1% (v/v) goat serum in phosphate buffered saline (GSPBS) and incubated with tissues overnight. Specifications and working dilutions for these antibodies are described in Table 1. The primary antibodies were detected with goat anti-rabbit secondary antibodies (British BioCell, UK) coated with a 1 nm gold particle diluted in GSPBS (1∶100) for 1–4 h at room temperature. The immunogold was visualized by silver intensification as described previously [39], [40], [42].

**Table 1:** Amino acid primary antibodies used in this study.

Antigen	Immunogen	Manufacturer, Cat No	Dilution
Aspartate	Aspartate conjugated to bovine serum albuminwith glutaraldehyde	Signature Immunologics (D100); Gift fromDr R. E. Marc	1∶400
Gamma-aminobutyric acid (GABA)	GABA conjugated to bovine serum albuminwith glutaraldehyde	Chemicon (AB5016); Signature Immunologics (YY100); Gift from Dr R. E. Marc	1∶4500
Glutamate	Glutamate conjugated to bovine serum albuminwith glutaraldehyde	Chemicon (AB5018); Signature Immunologics (E100); Gift from Dr R. E. Marc	1∶4500
Glutamine	Glutamine conjugated to bovine serum albuminwith glutaraldehyde	Chemicon (AB5012); Signature Immunologics (Q100); Gift from Dr R. E. Marc	1∶4000
Glycine	Glycine conjugated to bovine serum albuminwith glutaraldehyde	Chemicon (AB5020); Signature Immunologics (G100); Gift from Dr R. E. Marc	1∶4000
Taurine	Taurine conjugated to bovine serum albuminwith glutaraldehyde	Chemicon (AB5022); Signature Immunologics (TT100); Gift from Dr R. E. Marc	1∶1000

The antibodies used for this study were kindly donated by Dr R. E. Marc but are now also commercially available through Chemicon and Signature Immunologics. All antibodies are rabbit polyclonal.

Images were acquired using a LEICA DC500 camera, version 4.1.0.0 attached to a LEICA microscope (Leica Microsystems Ltd, Heerbrugg, Germany). All images were captured using fixed parameters of exposure time, intensity, contrast, gamma, brightness and eight-bit channel.

Toluidine blue stained upstream and downstream specimens were used in the drawing of schematics of the retina (Figure 1). For putative glycinergic interplexiform cells, serial 250 nm sections were stained for glycine and viewed with the aid of a *camera lucida* to determine the morphology of major glycine interplexiform cell dendrites.

Cell identification was based on anatomical classification described in previous studies of the lamprey retina [3], [4], [10], [30]. Briefly, cells were classified as photoreceptors, horizontal cells, bipolar cells, interplexiform cells or amacrine/ganglion cells based on retinal location and cell size. For bipolar cells, axon distribution was used to confirm identity. Amacrine and ganglion cells were grouped as a single class as the majority of the ganglion cells in the lamprey are displaced to the INL and cannot be differentiated from amacrine cells on anatomical features alone [12].

### AGB Incubation

Isolated eye cup pieces were mounted on 0.8 µm pore metrical membrane filters (Gelman Sciences, Ann Arbor, MI) and the sclera/retinal pigment epithelium removed by gently pulling it away from the retina. Retinal pieces were then incubated for 5–60 min at room temperature (∼20°C) under normal room lighting (300–400 lux) in a modified Edwards medium (125 mM NaCl, 2.5 mM KCl, 26 mM NaHCO_3_, 1.25 mM NaH_2_PO_4_, 10 mM dextrose, 2 mM CaCl_2_, 1 mM MgCl_2_, pH 7.4) bubbled with 95% O_2/_5% CO_2_ [43]. For tissue incubated with AGB, 25 mM AGB was added to the medium and an equimolar reduction in NaCl concentration was made. Retinal pieces were then fixed and processed for immunocytochemistry.

### Indirect Immunocytochemistry

Retinal pieces were fixed in 4% (w/v) paraformaldehyde, 0.01% (w/v) glutaraldehyde in PB for 30 min and then cryoprotected in 30% (w/v) sucrose overnight. Frozen vertical sections were collected on positively charged slides (Lomb Scientific, Taren Point, NSW, Australia). Tissues were blocked with 6% (v/v) goat serum, 1% (v/v) bovine serum albumin and 0.5% (v/v) Triton X-100 in PB for 1 h at room temperature followed by incubation with primary antibodies, diluted in 3% (v/v) goat serum, 1% (v/v) bovine serum albumin and 0.5% (v/v) Triton X-100 in PB overnight at 4°C. Details and dilutions of the primary antibodies used for indirect immunocytochemistry are provided in Table 2.

**Table 2:** Primary antibodies used in the immunofluorescence protocol.

Antigen	Immunogen	Manufacturer, Cat No	Host	Dilution
Agmatine (AGB)	Agmatine conjugated to bovine serum albumin	Chemicon; AB1568*	Rb; polyclonal	1∶100
Calbindin (CalB)	bovine kidney calbindin-D	Sigma-Aldrich; C9848	Ms; monoclonal	1∶1000
Calretinin (CalR)	Recombinant rat calretinin	Millipore; MAB1568	Ms; monoclonal	1∶1000
Glutamine synthetase (GS)	Recombinant sheep GS, amino acids1–373	BD Transduction Laboratories;610517	Ms; monoclonal	1∶3000
Protein kinase C-α (PKCα)	Purified bovine brain PKC	Sigma-Aldrich; P5704	Ms; monoclonal	1∶400
Recoverin	Recombinant human recoverin	Chemicon; AB5585	Rb; polyclonal	1∶1000

* This antibody was kindly donated by Dr R. E. Marc but is also commercially available through Chemicon.

Primary antibodies were detected with an anti-goat secondary antibody conjugated to AlexaFluor488 (Molecular Probes, Eugene, OR) diluted in 1∶500 in 3% (v/v) goat serum, 1% (v/v) bovine serum albumin and 0.5% (v/v) Triton X-100 in PB. The specificity of secondary antibodies was confirmed by omitting the primary antibody and using a secondary antibody from a different species. Fluorescence images were captured using a confocal laser scanning microscope (LEICA Microsystems TCS 4D). Image brightness and contrast was adjusted using Adobe Photoshop (version 6; Adobe Systems, Mountain View, CA, USA).

### Antibody Characterization

For the amino acid antibodies, the specificity of the glutamate antibody was previously shown in dot immunoassays where a positive signal for the glutamate antibody was obtained against an artificial glutamate antigen coupled to bovine serum albumin (BSA) via glutaraldehyde cross-linking [40]. Comparable results were observed for GABA, glycine, aspartate, taurine and glutamine antibodies against glutaraldehyde cross-linked GABA, glycine, aspartate, taurine and glutamine antigens respectively [40], [41], [44]. No cross-reactivity was observed with amino acids other than that of the antibody target (manufacturer’s data sheet, [40]). The specificity of the AGB antibody has been confirmed with dot blot immunoassays which report no cross reactivity with other amino acids [45]. No endogenous AGB activity has been detected in the vertebrate retina [45]–[47].

The specificity of the calbindin antibody was established in immunoblots yielding a single 28 kDa band corresponding to the expected calbindin protein (manufacturer’s data sheet). Specificity of the calretinin antibody has been confirmed by Western blots of mouse brain lysate which show the antibody reacting with a single 31 kDa band corresponding to the expected size for the calretinin protein (manufacturer’s data sheet). The specificity of the glutamine synthetase (GS) antibody was confirmed with a Western blot using rat cerebellum lysate where it reacted with a 45 kDa band which corresponds to the expected size of the GS protein, amino acids 1–373 (BD Biosciences technical data sheet). The specificity of protein kinase C-α (PKCα) antibody was confirmed in a Western Blot of rat glioma extract and NIH 3T3 mouse fibroblast lysate where the antibody reacted with a single 80 kDa band corresponding the expected size for PKCα. Finally, the specificity of the recoverin antibody was confirmed as the presence of single 26 kDa band in Western blots of human adult retina tissue homogenate [48].

## Results

### Retinal Anatomy of Downstream and Upstream Migrants

Figure 1 is a schematic representation of the lamprey retina based upon Nissl stained sections [49]. In contrast to many vertebrates, the majority of the ganglion cells in the lamprey are displaced to the INL and the nerve fibre layer is found between the INL and inner plexiform layer (IPL). A few orthotopic ganglion cells lie at the vitread border of the IPL and in the middle of the IPL. The downstream *G. australis* retina features 5 different cone-like photoreceptors and densely packed INL with over 10 layers of cells including at least three, possibly four layers of horizontal cells (S.P. Collin, unpublished data). The upstream *G. australis* retina is much thicker; dominated by large photoreceptors, some with an altered morphology (e.g. the C2 photoreceptor replaces a photostable pigment with an unpigmented ellipsosome). The number of cell layers in the INL appears reduced but four layers of horizontal cells are visible. Similar to other vertebrates, the lamprey retina has a pigmented epithelium.

### Glutamate Immunoreactivity of *G. australis* retina

**Figure 2:**
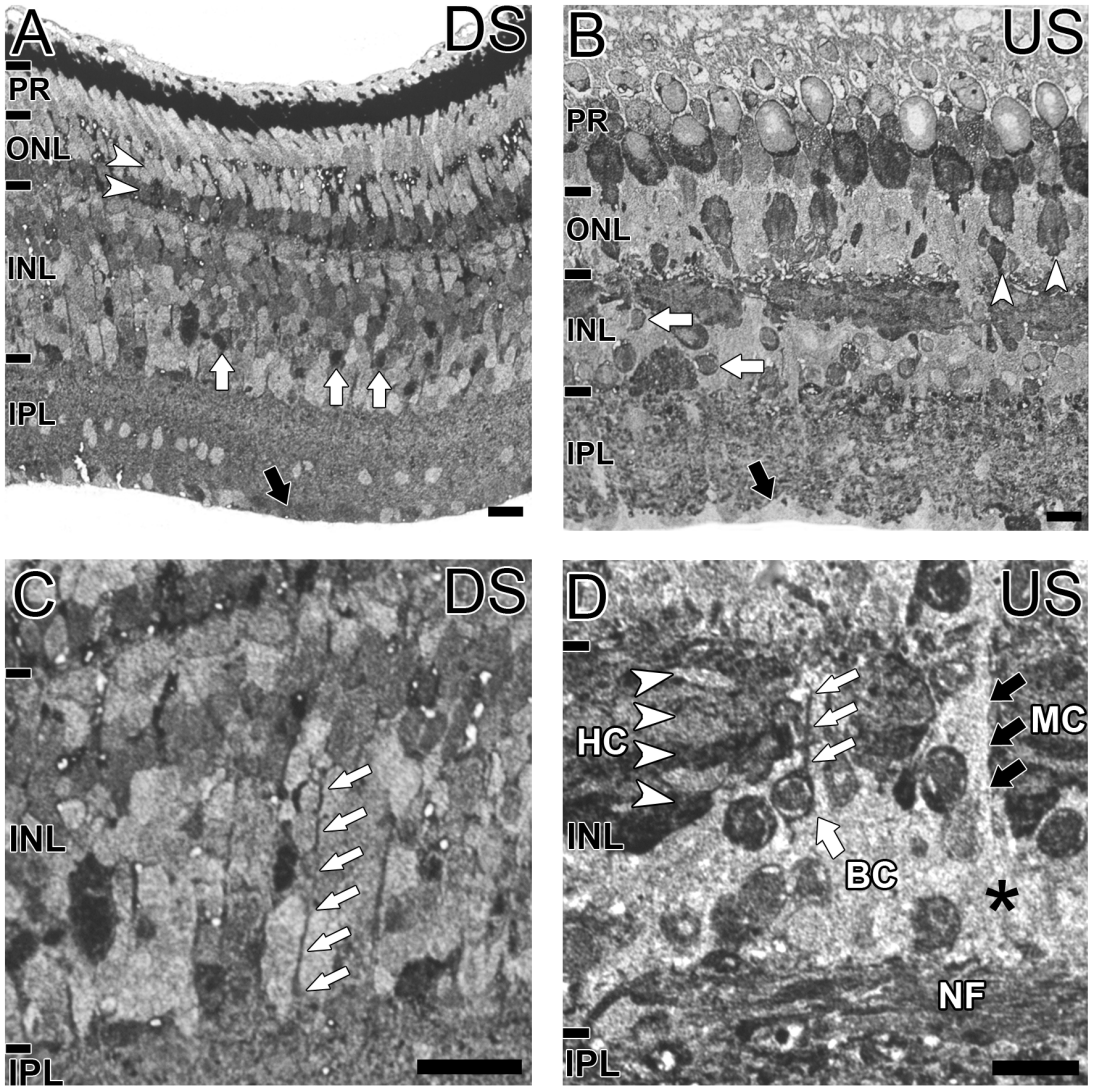
Localization of glutamate in the G. australis retina. Glutamate immunoreactivity in the **A, C**: downstream migrant (DS) and **B, D**: upstream migrant (US) of *G. australis.* **A:** Glutamate immunoreactivity in the downstream migrant was observed in the ONL at different intensities (white arrowheads). Strong immunoreactivity was also seen in some cells in the INL (white arrows) and Müller cell endfeet (black arrow). **B:** In the upstream migrant, numerous large and small photoreceptor cells were immunoreactive (white arrowheads), as well as other INL cells (white arrows). Müller cell endfeet displayed low glutamate immunoreactivity (black arrow). Magnified images confirm that **C:** Müller cells in the downstream migrant were immunoreactive for glutamate (white arrows) and **D:** horizontal cells (HC; white arrowheads), putative bipolar cells (BC; white arrows) and the nerve fiber layer (NF) are glutamate immunoreactive in the upstream migrant. Müller cell (MC) somata (asterisk) and processes (black arrows) were immunonegative. Retinal layers are denoted by the lines and annotations on the left of each image and include the photoreceptors (PR), outer nuclear layer (ONL), inner nuclear layer (INL) and inner plexiform layer (IPL). Note that due to the small thickness of the OPL, it was not labelled for clarity. Scale bar for all images is 20 µm.

Glutamate immunoreactivity was observed to some degree in all retinal cells of the downstream and upstream migrant of *G. australis* (Figure 2). In the downstream migrant, many cell somata were labelled in the outer nuclear layer (ONL) and there was a clear division between the weakly stained cells in the outer ONL and strongly stained cells in the inner part of the ONL (Figure 2A, white arrowheads). For the upstream migrant, at least two different photoreceptor types were labelled in the ONL; a large soma type in the middle of the ONL and a small cell soma located close to the ONL-OPL border (Figure 2B, white arrowheads). The inner nuclear layer (INL) also showed heterogeneous labelling with high levels of glutamate immunoreactivity in the outer INL. Glutamate was uniformly distributed across the horizontal cell layer for both animals except for disruptions by the Müller cell processes (Figure 2D, black arrows).

Many cells in the inner INL of the downstream migrant of *G. australis* showed a moderate level of staining but some were intensely stained (Figure 2A, white arrows). These cells could not all be confirmed as amacrine cells as many lamprey ganglion cells are displaced to the inner INL [12]. Glutamate immunoreactive cells processes traversing the INL appeared to display Müller cell morphological characteristics (Figure 2C, white arrows). The most proximal part of the retinal, where Müller cell endfeet terminate, was glutamate immunoreactive (Figure 2A, black arrow). In the upstream migrant, several cell types in the INL were strongly glutamate immunoreactive (Figure 2B, white arrows). Müller cell endfeet in the most proximal part of the retina display low glutamate (Figure 2B, black arrow) and Müller cell somata (Figure 2D, asterisk) display low levels of glutamate. Figure 2D highlights a labelled cell with is axon extending towards the outer retina suggesting bipolar cells are glutamate immunoreactive. Four layers of glutamate immunoreactive horizontal cells were also clearly evident (Figure 2D, white arrowheads). We performed PKCα labelling to further confirm bipolar cell identity but did not yield any distinct soma labelling in the retinae of downstream or upstream migrants (Figure S1).

### GABA Immunoreactivity of *G. australis* Retina

**Figure 3:**
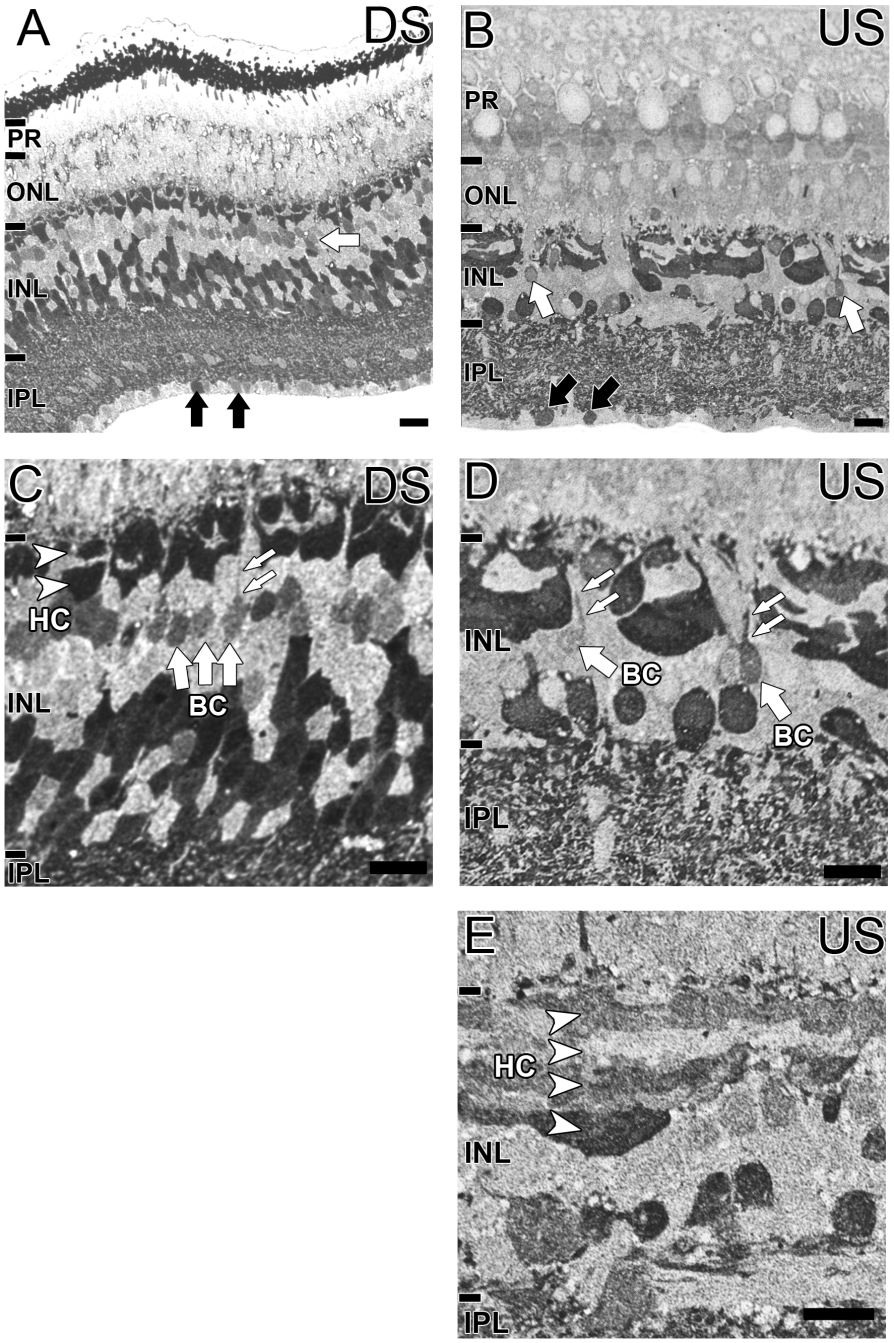
Localization of GABA in the G. australis retina. GABA immunoreactivity in the **A, C:** downstream migrant and **B, D, E:** upstream migrant of *G. australis.* **A–B:** GABA immunoreactivity of the whole retina. White arrows indicate weakly GABA positive bipolar cells and black arrows indicate GABA reactive cells in the IPL. **C–E:** Magnified images of the INL. White arrowheads indicate GABA immunoreactive horizontal cells (HC) and white arrows in highlight bipolar cell (BC) processes extending towards the outer plexiform layer. Abbreviations are as in Figure 2. Scale bar is 20 µm for all images.

GABA immunoreactivity was almost identical between downstream and upstream migrating animals, restricted mostly to the inner retina (Figure 3). A band of weakly GABA immunoreactivity cells were evident in the outer INL of the downstream migrant (Figure 3A, white arrow) and more sparingly in the upstream migrant (Figure 3B, white arrows). Closer inspection suggests these cells as bipolar cells with evidence of processes extending into the outer plexiform layer (OPL; Figure 3C–D). At least two distinct layers of highly immunoreactive horizontal cells were evident in the downstream migrant (Figure 3C, white arrowheads) and three layers of horizontal cells were strongly GABA immunoreactive in the upstream migrant (Figure 3E, white arrowheads). Most cells of the inner INL were strongly labelled for GABA in both animals. Intense GABA labelling was also present in the IPL of both animals and some cells along the vitreous IPL border (Figure 3A–B, black arrows). In both upstream and downstream migrant retinas, the most proximal retina showed low GABA immunoreactivity indicating Müller cell endfeet do not have constitutively high levels of this amino acid.

### Glycine Immunoreactivity of *G. australis*

**Figure 4:**
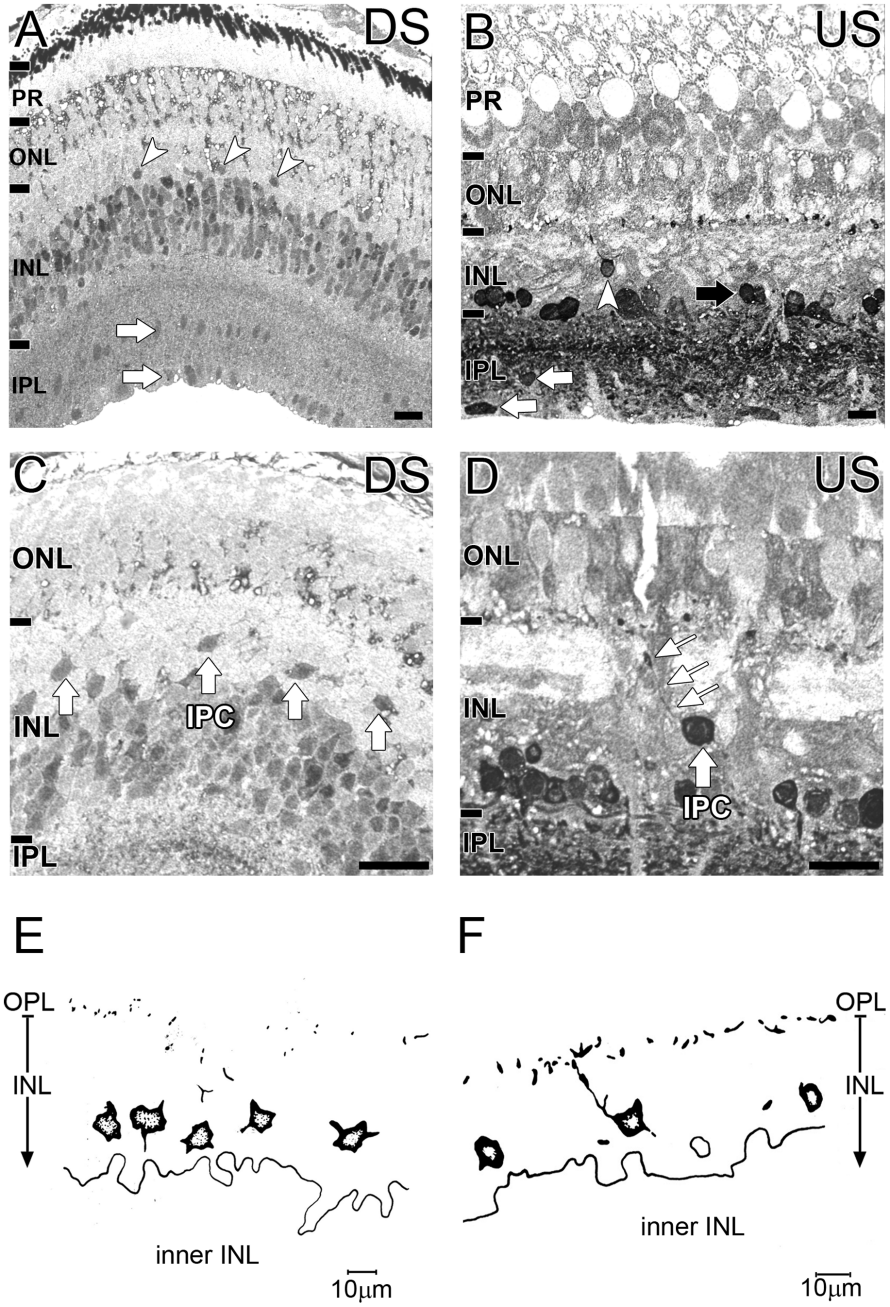
Localization of glycine in the G. australis retina. Glycine immunoreactivity of **A, C, E:** the downstream migrant and **B, D, F:** upstream migrant of *G. australis.* **A–B:** Glycine immunoreactivity of total retina where white arrows indicate labelled cells in the mid and inner IPL and white arrowheads indicate strongly labelled putative interplexiform cells (IPC). **C–D:** Magnified images of putative glycine immunoreactive IPCs (white arrows). **E–F:** The morphology of putative glycinergic interplexiform cells and their dendrites drawn with the aid of a camera lucida. Abbreviations are as in Figure 2. Scale bar is 20 µm in microscope images and 10 µm in drawings.

Glycine was mostly restricted to the inner retina of *G. australis* (Figure 4). In the downstream animal, glycine immunoreactive cells were present in the inner INL and displayed varied levels of labelling (Figure 4A). Some glycine immunoreactivity was also evident in the outer retina. Glycine immunoreactive cells of the upstream migrant however were more concentrated along the INL - IPL border (Figure 4B, black arrow). Additionally, intense glycine reactive processes were observed throughout the IPL of the upstream migrant of *G. australis* but not the downstream migrant. Glycinergic cells in the mid and inner IPL were observed in the both animals (Figure 4A–B, white arrows).

A few isolated cells in the outer INL were also strongly glycine immunoreactive in both animals (Figure 4A–B, white arrowheads). These cells are thought to be putative glycinergic interplexiform cells with evidence of the major processes extending towards the IPL and OPL (Figure 4C–F). The punctate glycine immunoreactivity seen in the OPL is likely to reflect their cell processes.

### Glutamine Immunoreactivity of *G. australis* Retina

**Figure 5:**
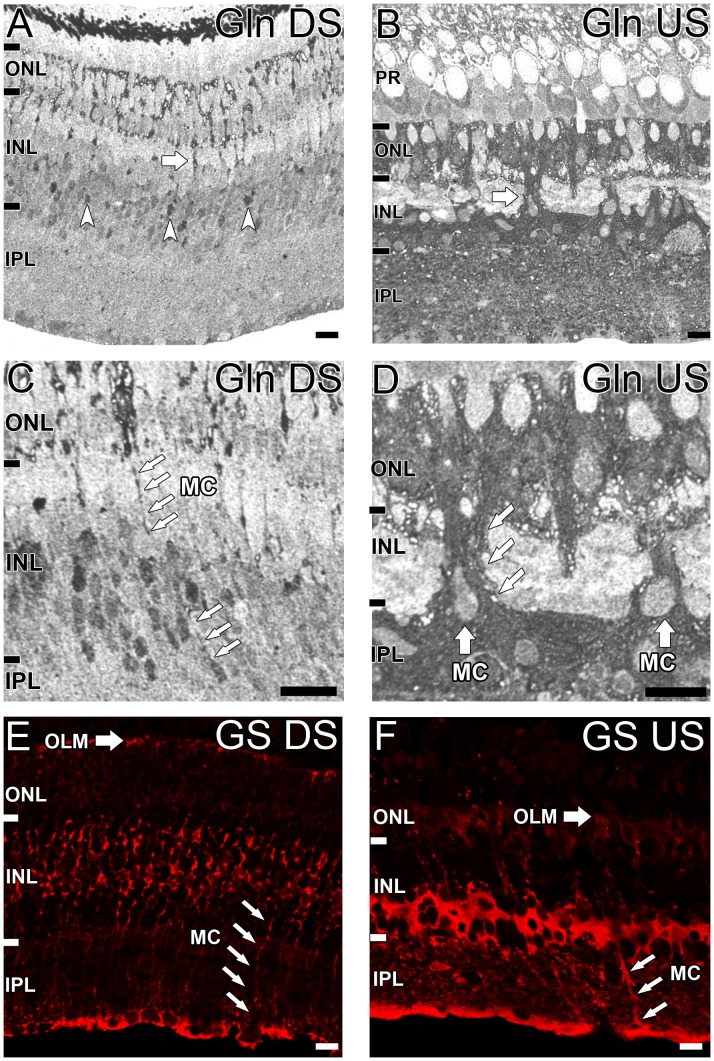
Localization of glutamine in the G. australis retina. Glutamine (Gln) immunoreactivity in the **A, C:** downstream migrating (DS) and **B, D:** upstream (US) migrating *G. australis.* **A–B:** Glutamine immunoreactivity across the whole retina. White arrowheads indicate the few immunoreactive cell somata in the INL and white arrows indicate the columns of glutamine immunoreactivity extending towards the ONL. **C–D:** Magnified images of the INL demonstrating Müller cell processes traversing the retina (white arrows). Glutamine synthetase (GS) immunoreactivity was also determined in the **E:** downstream and **F:** upstream migrating *G. australis.* Müller cell (MC) processes are indicated with small white arrows and the immunoreactive outer limiting membrane (OLM) but a large white arrow. Abbreviations are as in Figure 2. Scale bar is 20 µm for all images.

Glutamine immunoreactivity differed considerably between the migratory phases of *G. australis*. Weak glutamine immunoreactivity was observed across the retina of the downstream migrant except for few intensely stained somata observed in the mid INL (Figure 5A, white arrowheads). These cells are likely to be Müller cells as magnified images show columns of glutamine immunoreactivity corresponding to typical pattern of Müller cell processes traversing the ONL (Figure 5A, C, white arrows). Glutamine synthetase (GS) immunoreactivity also closely matched glutamine immunoreactivity, present as punctuate labelling along the outer limiting membrane (OLM; Figure 5E, large white arrow) and immunoreactive processes extended across the entire retina (Figure 5E, small white arrows).

Glutamine immunoreactivity was more extensive in the upstream migrating *G. australis,* found throughout the outer and inner retina. Immunoreactivity was mostly concentrated to the surrounding cell matrix with many cell somata remaining immunonegative, particular horizontal cells (Figure 5B). Thick columns of glutamine immunoreactivity could be seen traversing the horizontal cell layer into the ONL (Figure 5D, white arrows). Somata at the base of these columns were likely Müller cells. Increased levels of GS labelling was also observed in the upstream migrant of *G. australis* (Figure 5F). The similarity of glutamine synthetase and glutamine immunoreactivity suggests the same cell type is being labelled.

### Aspartate Immunoreactivity of *G. australis* Retina

**Figure 6:**
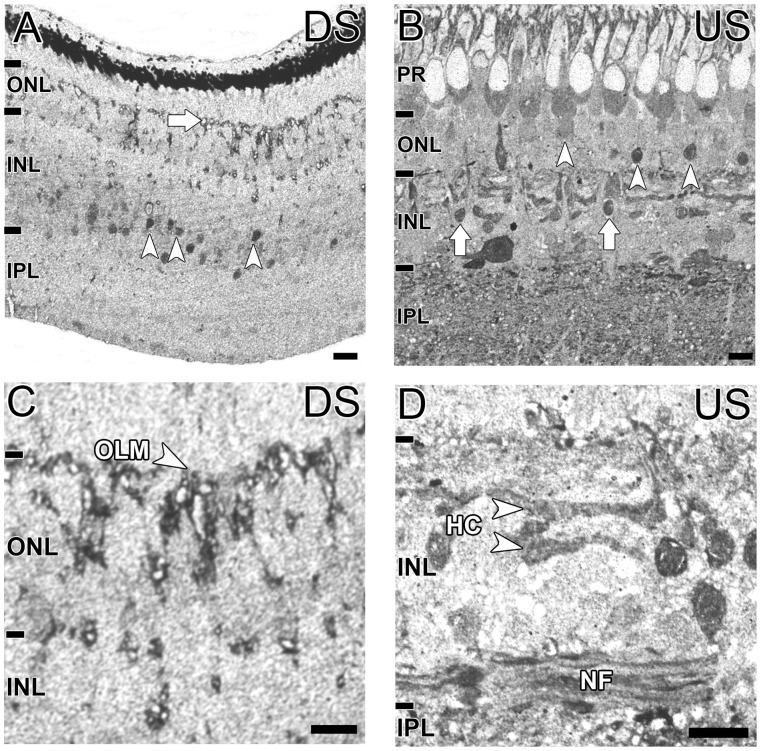
Localization of aspartate in the G. australis retina. Aspartate immunoreactivity in **A, C:** the downstream migrant and **B, D:** the upstream migrant of *G. australis.* **A:** Aspartate immunoreactivity for the downstream animals was confined to the mid INL (white arrowheads) and punctate staining along the outer limiting membrane (white arrow). **B:** Aspartate immunoreactivity in the upstream animal was observed in different photoreceptors (white arrowheads) and some small cells in the INL (white arrows). **C:** Magnified image of the downstream migrant retina showing aspartate immunoreactivity across the outer limiting membrane (OLM). **D:** Magnified image of the INL in the upstream migrating *G. australis* showing immunoreactive horizontal cells (HC) and nerve fibers (NF) are indicated. Abbreviations are as in Figure 2. Scale bar is 20 µm in all figures.

In the downstream animal, aspartate was limited to a few cells in the mid and inner INL, similar to the immunoreactivity profile of glutamine (Figure 6A, white arrowheads). High aspartate immunoreactivity at the OLM also suggests the labelling of Müller cell microvilli (Figure 6C, white arrowhead). On the other hand, many cell types contained aspartate in the upstream migrating *G. australis.* In the ONL, a cell population near the photoreceptor layer and another near the ONL-OPL border were immunoreactive (Figure 6B, white arrowheads). Within the INL, some small cells in the outer INL (possibly bipolar cells) and a few cells at the INL-IPL border were labelled (Figure 6B, white arrows). Aspartate was also present in a number of horizontal cells and nerve fibers (Figure 6D, white arrowheads). Asparate labelled horizontal cells were co-localized with glutamate and GABA immunoreactivity (M. Kalloniatis, unpublished data).

### Taurine Immunoreactivity of *G. australis* Retina

**Figure 7:**
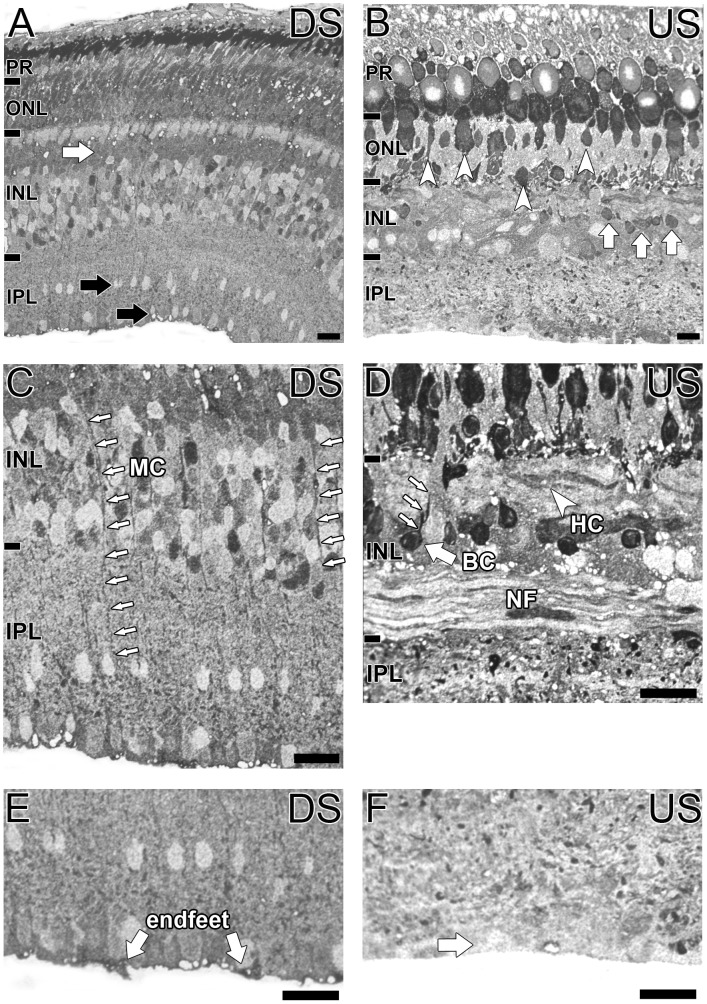
Localization of taurine in the G. australis retina. Taurine immunoreactivity in **A, C, E:** the downstream migrant and **B, D, F:** the upstream migrant of *G. australis.* **A–B:** Taurine immunoreactivity across the whole retina length. White arrows indicate putative taurine labelled bipolar cells, black arrows indicate weakly labelled IPL cells and white arrowheads indicate various photoreceptor types immunoreactive for taurine. **C:** Magnified image of the INL with white arrows indicating immunoreactive Müller cell processes. **D:** Magnified image of the INL in the upstream migrant of *G. australis.* Taurine immunoreactivity was present in a putative bipolar cell (BC; white arrows) and a weakly labelled horizontal cell (HC). Immunoreactivity was absent in nerve fibers (NF). **E–F:** Taurine immunoreactivity of vitreous-IPL border where Müller cell endfeet are located. Abbreviations are as in Figure 2. Scale bar is 20 µm.

High levels of taurine were present throughout the downstream and upstream migrating *G. australis* retina particularly in the photoreceptors (Figure 7A–B). In the upstream migrant of *G. australis,* several photoreceptors types with varying sized somata were clearly labelled (Figure 7B, white arrowheads). Highly taurine immunoreactive cells were also obvious in the outer INL of both animals (Figure 7A–B, white arrows). In the upstream migrant of *G. australis,* these cells appeared to be bipolar cells with processes extending towards the outer retina, similar to glutamate labelled processes (Figure 7D, white arrows). We were unable to find distinct taurine labelled cell processes in the downstream migrant although their anatomical location suggests they were also bipolar cells. All horizontal cells of the downstream migrant had low taurine immunoreactivity whilst one horizontal layer in the upstream migrant was weakly taurine immunoreactive (Figure 7D, white arrowhead). Cells of the inner INL and IPL of both migrants were mostly immunonegative (Figure 7A, black arrows).

Interestingly, taurine immunoreactivity of Müller cells differed in the upstream and downstream migrant. Taurine immunoreactive cell processes could be seen throughout the retina of the downstream migrant and highly immunoreactive Müller cell endfeet were obvious at the vitreal IPL border (Figure 7C, E, white arrows). In contrast, we saw no obvious taurine labelling of Müller cell processes in the upstream migrant of *G. australis* and very little immunoreactivity was evident along the IPL-vitreous border (Figure 7F, white arrows). The large Müller cell and taurine immunoreactive somata (Figure 7B), display similar morphology to the glutamine synthetase and glutamine immunoreactivity.

### Cation Channel Permeability of Retinal Neurons in *G. australis*

**Figure 8:**
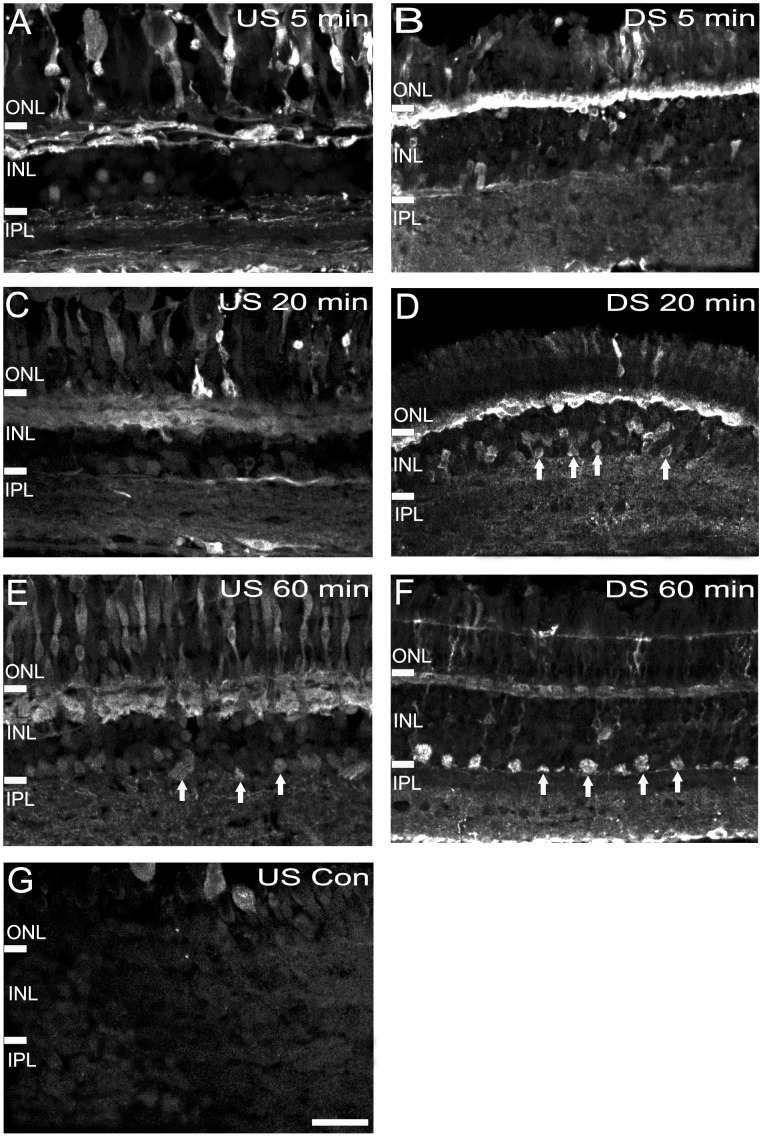
Cation channel permeability based on AGB immunoreactivity. Retinas of downstream (DS) and upstream (US) migrating *G. australis* were incubated for **A–B:** 5 min, **C–D:** 20 min, **E–F:** 60 min and **G:** 0 min with AGB. AGB labelled cells in the INL are indicated with white arrows. Abbreviations are as in Figure 2. Scale bar is 20 µm.

AGB is a small organic cation and can be used to map excitatory pathways regulated by glutamate activity in the retina [45], [50]–[52]. We assessed basal cation channel permeability in upstream and downstream migrating animals using AGB. After a 5 min incubation with AGB, several photoreceptors and horizontal cells were strongly AGB immunoreactive in both upstream and downstream migrants of *G. australis* (Figure 8A–B). Weakly labelled cell bodies in the inner retina were also apparent. Longer incubation times (20–60 min) resulted in a successive increase in the number of AGB labelled cell somata in the INL (Figure 8C–F). In the downstream migrating *G. australis*, AGB immunoreactive neurons were distributed throughout the inner half of the INL (Figure 8D, white arrows). By 60 min, the strongest AGB immunoreactivity was localized to cells along the INL-IPL border and inner IPL (Figure 8F, white arrows). Distinct AGB immunoreactivity in the INL was not as obvious in the retina of the upstream migrating *G. australis* although many AGB labelled cells along the INL-IPL border could be seen after 60 min (Figure 8E, white arrows). Interestingly, no AGB was observed for cells in the mid IPL in both migrant animals. Many photoreceptors and all horizontal cells were strongly immunoreactive by the end of the AGB time course. Very little endogenous AGB was present in the *G. australis* retina (Figure 8G).

### Distribution of Calcium Binding Proteins

**Figure 9:**
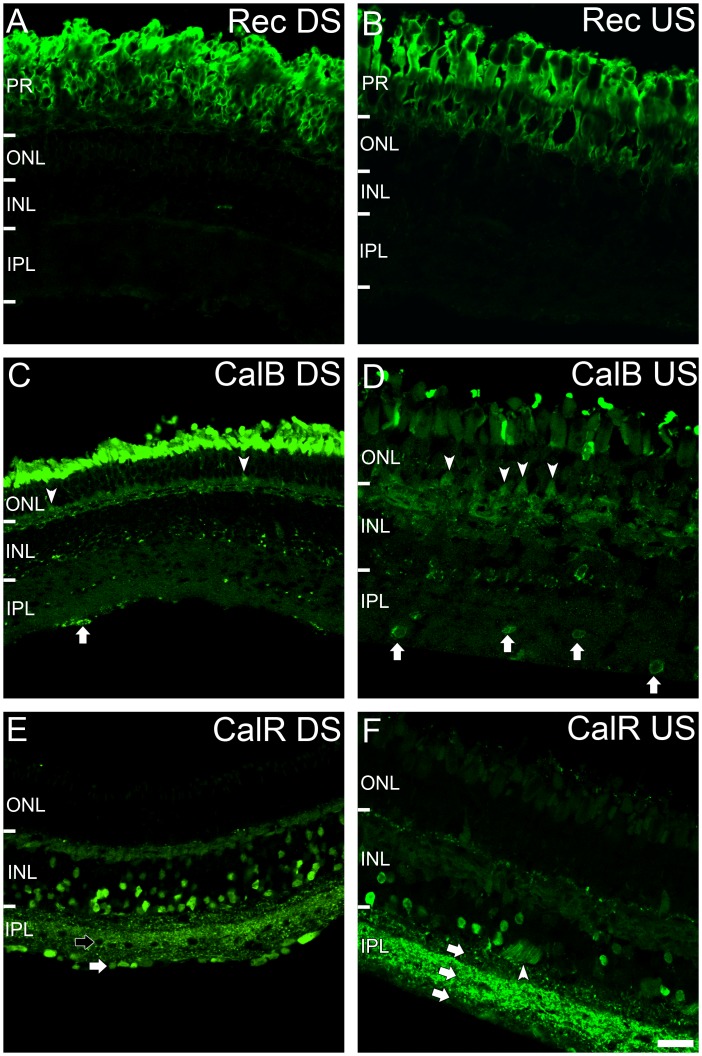
Distribution of calcium binding proteins in the retina of *G. australis*. Recoverin (Rec) immunoreactivity in the **A:** downstream (DS) migrating and **B:** upstream (US) migrating *G. australis.* Calbindin (CalB) immunoreactivity in the **C:** downstream migrating and **D:** upstream migrating *G. australis* ***.*** Labelled photoreceptors and IPL cells are denoted by white arrowheads and arrows respectively. Calretinin (CalR) immunoreactivity in the **E:** downstream migrating and **F:** upstream migrating *G. australis* ***.*** In the downstream migrating animal (**E**), CalR immunoreactive cells in the inner IPL were found near the inner limiting membrane (white arrow) but not in the mid IPL (black arrow). In the upstream migrating animal (**F**), three different bands of CalR intensity was present in the IPL (white arrows). The white arrowhead indicates labelled nerve fibers. Abbreviations are as in Figure 2. Scale bar is 50 µm.

Calcium ions play a key role alongside amino acid neurotransmitters in signal transduction in the retina. Hence, we investigated the distribution of the calcium binding proteins, calbindin (CalB), calretinin (CalR) and the calcium sensitive protein, recoverin, to analyse calcium associated protein distribution in the retina of *G. australis*. Recoverin immunoreactivity was identical between the downstream and upstream migrants of *G. australis*, which was localized completely to photoreceptors (Figure 9A–B).

CalB immunoreactivity in the retina of the downstream migrating *G. australis* was found in the outer segment/inner segment of photoreceptors, horizontal cells and in the inner INL (Figure 9C). Staining, however, was fairly diffuse and individual cell bodies were difficult to distinguish. A triangular shaped cell body labelled in the ONL suggests that some photoreceptors may be CalB positive (Figure 9C, white arrowhead). Punctate staining present in the mid IPL suggested cells of the INL express CalB. A labelled cell soma in the inner IPL was also apparent (Figure 9C, white arrow). Immunoreactivity of the upstream migrant retina was similar to its downstream counterpart. All horizontal cells were CalB immunoreactive and some cell bodies in the inner INL were labelled. A number of CalB immunoreactive photoreceptors were evident in the ONL (Figure 9D, white arrowheads). Some isolated cells in the mid and inner IPL were also CalB immunoreactive (Figure 9D, white arrows).

CalR immunoreactivity was more conspicuous than CalB, distinctly labelling several cell somata in the inner retina (Figure 9E–F). For the downstream migrant, weak CalR staining was observed throughout the horizontal cell layer and several bipolar cells in the outer INL. Many cells in the inner INL were also labelled. Positive staining was seen for cells in the inner IPL (Figure 9E, white arrow) but absent in cells in the mid IPL (Figure 9E, black arrow). This pattern of immunoreactivity was matched in the upstream migrating *G. australis* with weak CalR immunoreactivity present in outer INL and strong labelling of the inner INL (Figure 9F). Labelling of nerve fibers (Figure 9F, white arrowhead) suggests that ganglion cells expressed CalR. Punctate staining was evident throughout the IPL, distributed into three distinct layers; an intensely stained middle layer surrounded by two layers of moderate CalR immunoreactivity (Figure 9F, white arrows).

## Discussion

### Glutamate Distribution in the *G. australis* Retina

**Figure 10:**
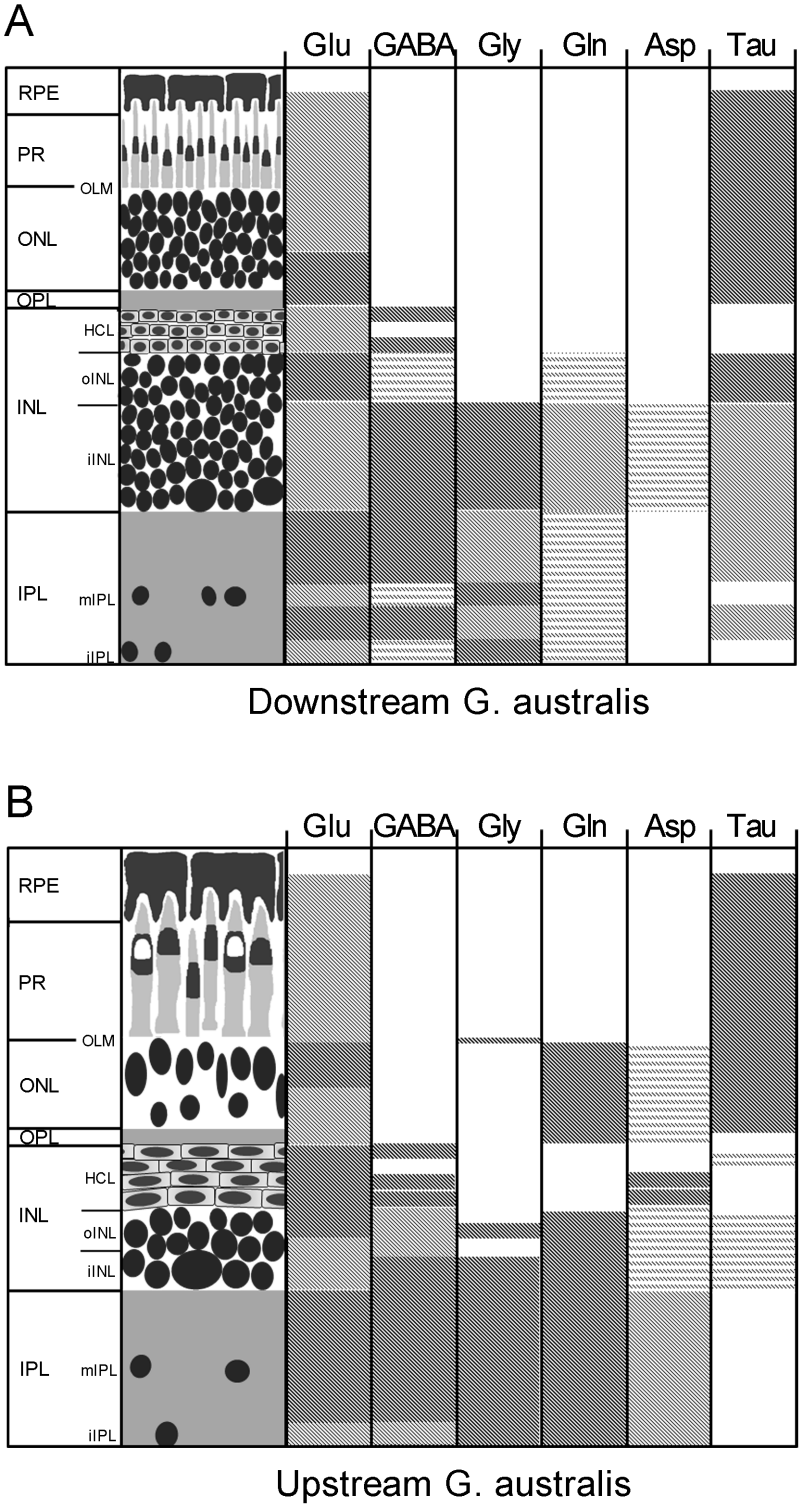
Schematic of amino acid distribution in the retina of *G. australis*. Schematic of the **A:** downstream migrant and **B:** upstream migrant retinas and their relative amino acid distribution. Drawings on the left indicate the retinal organisation and the degree of shading indicates the average intensity of labelling seen for that amino acid relative to labelling in the rest of the retina. Abbreviations – Glutamate (Glu), γ-aminobuytric acid (GABA), glycine (Gly), Glutamine (Gln), Aspartate (Asp), Taurine (Tau). All other abbreviations are as in Figure 1.

Glutamate is the major excitatory neurotransmitter in the retina [39], [40], [53]–[56]. Our results found glutamate in virtually all retinal neurons of *G. australis* with high levels in photoreceptors, horizontal, amacrine and ganglion cells. A summary of amino acid distribution in the retina of *G. australis* is shown in Figure 10. Glutamate immunoreactivity in the inner retina matched patterns in the northern hemisphere lamprey, *P. marinus,* and several other vertebrates including the goldfish, rat, chicken, cat, monkey and human [22], [39], [41], [54], [56]–[60]. Glutamate was not detected in photoreceptors of postmetamorphic individuals of other lamprey species [22]. This may be due to different photoreceptor morphologies of northern and southern hemisphere lamprey species [3], [30]. Indeed, glutamate immunoreactivity of photoreceptors of other vertebrates demonstrate considerable diversity [61]. Alternatively, differences in experimental methodology may play a role. This was noted in studies with primate retina, where differences in tissue fixation and manipulation lead to significant changes in amino acid distribution post-mortem [59], [60], [62], [63]. Glutamate immunoreactive photoreceptors in the downstream migrant of *G. australis* also showed a unique distribution with higher glutamate levels near the OPL. This may be due to the uptake of glutamate from the OPL by photoreceptors to deactivate neurotransmission [64], [65].

### GABA and Glycine Distribution in the *G. australis* Retina

The major inhibitory neurotransmitters-GABA and glycine, are commonly associated with lateral retinal elements. GABA is particularly dominant in amacrine cells and shows variable expression in horizontal cells [41], [56], [58], [59], [61], [66]–[68]. For *G. australis*, strong GABA immunoreactivity was observed in horizontal and amacrine cells at both migratory phases. GABA negative cells in the INL were potentially glycinergic amacrine cells or displaced ganglion cells as GABA is typically absent in ganglion cells [40], [69]. Similarly, poor GABA immunoreactivity of cells of the IPL suggests this population contains orthotopic ganglion cells or glycinergic displaced or interstitial amacrine cells [3], [22], [70]. Weak GABA immunoreactivity present in putative *G. australis* bipolar cells matched other lamprey species [22], amphibians [66], [71], [72] and some mammals [69], [73]–[76]. Ascribing a specific bipolar cell type to this population however is difficult as few common features exist between GABAergic bipolar cells in different species. For example, GABA is localized specifically to rod bipolar cells in the primate retina [59], [74] whilst in the cat, GABA is present in two OFF cone bipolar cell types [77]. Lack of consistency across species suggests GABA bipolar cells are a species-specific adaptation rather than a fundamental feature of vertebrate visual system evolution.

Glycine is also predominantly found in amacrine cells [61], [69], [78]–[82] and to a lesser extent, bipolar cells due to amacrine cell coupling [78], [83]–[85]. We saw highly glycine immunoreactive interplexiform cells in the INL of both upstream and downstream migrating *G. australis*. These cells have been found in teleost fish [86]–[88], amphibians [89], birds [39] and other lamprey species [22] but not in mammals. Glycine interplexiform cells synapse with horizontal and amacrine cells indicating a role in feedback between the outer and inner retina [90]–[93]. Glycine immunoreactive cells in the inner INL probably correspond to amacrine cells rather than displaced ganglion cells. Most cells in the IPL were also glycine immunoreactive. In the lamprey *P. marinus,* this population was described as displaced amacrine cells as glycine is rarely detected in vertebrate ganglion cells [39], [59], [60], [69]. However, Marc and Jones (2002) showed ganglion cells may contain glycine through gap junctions with other glycinergic cells [94]. The glycine immunoreactive IPL cells were also anatomically similar to interstitial glycinergic amacrine cells observed in the IPL of the goldfish, shark and lizard retinas [61], [70]. Low levels of GABA immunoreactivity in these cells further support classification of this population as amacrine cells [70]. Finally, glycine observed in the outer retina of the downstream migrant corresponded to typical pattern of Müller cell processes. Indeed, a unique feature of Müller cells of the lamprey retina is a high affinity uptake system to deactivate glycine [88].

### Glutamine and Aspartate Distribution in the *G. australis* Retina

Glutamine and aspartate play metabolic roles in the retina, maintaining amino acid homeostasis as precursors for glutamate [95], [96]. Glutamine is mostly found in Müller cells [39], [41], [59] as well as ganglion cells and horizontal cells in some vertebrates [28], [41], [54], [56], [59], [97], [98]. For *G. australis*, glutamine was confined to Müller cells, notable by columns of strong glutamine immunoreactivity in the intercellular space of the outer and inner nuclear layers which closely matched glutamine synthetase labelling. In the horizontal layer, glutamate and GABA immunoreactivity was clearly absent in regions corresponding to Müller cell processes, suggesting that rapid uptake and breakdown of neurotransmitters is a primitive feature of Müller cells [96]. Between migratory phases, glutamine levels were considerably greater in the upstream migrating *G. australis* than the downstream animal. This was closely coupled with increased glutamine synthetase immunoreactivity indicating growth of Müller cells in response to retinal adaptation to the oceanic environment. Alternatively, differences could represent changes in glutamate/glutamine metabolism in glial cells due to changes in neurotransmission between migrants.

Aspartate immunoreactivity also showed dramatic changes - isolated to a few cells in the INL of the downstream migrating *G. australis* compared to extensive immunoreactivity in photoreceptors, horizontal cells and the INL in the upstream migrant. In other lamprey species, aspartate immunoreactivity was absent in the larval stages but present in putative ganglion cells, amacrine cells and horizontal cells in the adult animal [22]. Aspartate also co-localized with glutamate and GABA in some horizontal cell layers like some horizontal cell sub-populations in the chicken, peccary and lizard retinas [39], [56], [99]. Expansion of aspartate to the outer retina in the upstream migrant may reflect the need for large pools of neurotransmitter precursor to accommodate the metabolic requirements of the larger photoreceptors and horizontal cells [30]. Indeed, increased aspartate immunoreactivity in the chicken retina subjected to anoxic conditions indicates the sensitivity of this amino acid to the metabolic state of the retina [39], [100].

### Taurine Distribution in the *G. australis* Retina

Taurine has been implicated in osmoregulation, photoprotection, development and apoptosis of retinal neurons [41], [101]–[103]. Taurine was abundant in the *G. australis* retina, particularly in photoreceptors. Extensive taurine distribution is also present in the retinas of goldfish, cat, lizard, shark, monkey and rat supporting its role in fundamental cellular functions [41], [59], [61], [104]. Taurine immunoreactivity was also conserved between migratory phases except in Müller cells where taurine immunoreactive cell processes and endfeet were not visible in the upstream migrant. The South African clawed frog also displays little taurine labelling within Müller cells suggesting a possible link between metamorphosis and taurine immunoreactivity of glial cells [61].

### Amino Acid Neurochemistry and Retinal Anatomy during *G. australis* Migration

The anatomical changes which occur between the retinas of the downstream and upstream migrants of *G. australis* have been noted in several studies [3], [30], [32], [38]. Collin et al. (2003) showed that photoreceptors of the upstream migrant of *G. australis* are almost three times larger than equivalent photoreceptor types in the downstream migrant [30]. Additionally, peak spectral sensitivity of the cone-like photoreceptor C2, shifts in the upstream migrant of *G. australis* as the yellow myeloid pigment in the outer segment is replaced by a large unpigmented ellipsosome [3], [30]. Comparable changes are described for the retina of *P. marinus* [17] and metamorphosing individuals of fish and amphibian species which migrate between fresh water and oceanic environments [105], [106]. For example, upstream migrants of the European eel *Anguilla anguilla* show increased eye size, increased photoreceptor size compared to its downstream migrating counterpart [105]. Current theories point to increased eye size and photoreceptor changes in the lamprey as a response to selective pressures whereby animals in the ocean adopt retinal features which support scotopic vision to avoid avian predators at the water surface [3], [30]. This study now shows that Müller cells morphology is also altered in the lamprey, with the expansion of Müller cells in the upstream migrant of *G. australis*. Retinal amino acid neurochemistry also changes, particularly for glutamine and aspartate which are increased in the Müller cells of the upstream migrant (Figure 10). Altered Müller cell morphology and amino acids associated with glutamate production is possibly a response to the higher metabolic demands of the larger, reorganised upstream migrant retina. Thus, tight coupling of neuron activity and supporting glia is a feature of the early vertebrate retina.

### Cation Channel Permeability

Basal cation channel permeability of AGB was observed mainly among photoreceptors. Longer incubation times resulted in permeability into inner retinal neurons corresponding to patterns in the rabbit, where bipolar, horizontal, amacrine and some ganglion cells were labelled after a 20 min AGB incubation and the rat, where numerous inner retinal neurons were labelled after a 30 min AGB incubation [107], [108]. High levels of AGB were seen in all *G. australis* photoreceptor types. This is likely explained by the presence of only cone-like photoreceptors in the retina of *G. australis* as AGB permeability is thought to be restricted to cones through non-specific cation channels that are not modified with classic cation pharmaceuticals [45]. AGB permeability was similar between upstream and downstream migrating *G. australis* across the incubation time course. Previous studies in jawed vertebrates indicate that AGB enters cells through non-selective ion channels gated by ionotropic glutamate receptors [45]. Thus, identical AGB profiles coupled with similarities in glutamate immunoreactivity indicate that glutamate signalling does not change as the animal matures.

### Calcium Binding Protein Distribution

Recoverin immunoreactivity was confined to photoreceptors, corresponding to patterns observed in other species of lamprey [16] and jawed gnathostomatous vertebrates [109] and suggesting that this protein is highly conserved in the vertebrate visual system. CalB was evident in horizontal cells and amacrine and/or ganglion cells whilst CalR was apparent in bipolar, horizontal, amacrine and ganglion cells. Few differences were observed in CalB and CalR immunoreactivity between the upstream and downstream migrants. CalR negative cells, present in the mid-IPL of the retina of downstream migrant *G. australis*, appeared to be lost in the upstream migrant although this was possibly a product of the decreased cell density associated with an increase in eye size in the postmetamophic lamprey [17]. CalB and CalR immunoreactivity matched that of other lamprey species (*Petromyzon marinus*, *Lampetra fluviatilis*) suggesting conservation across species [18], [19]. Although some discrepancies are evident (i.e. horizontal cells of *L. fluviatilis* are CalB immunonegative), these differences probably reflect acquired species-specific variations rather than stages in evolutionary development. Unique to *G. australis* was the presence of CalB in photoreceptors. Although absent in other lampreys, CalB is present specifically in the cones of jawed vertebrates including pig, sheep, chick, cat and salamander [110]. Anatomical and functional data suggests that all five photoreceptor types in *G. australis* are cone-like, whilst those of other lamprey species are less defined, which may explain the discrepancies in CalB immunoreactivity [3], [26], [27]. Also interesting were striations of CalR immunoreactivity in the *G. australis* IPL. ChAT and serotonin immunoreactive striations in the IPL have also been observed [21], [24].

### Conclusion

We found the distribution of key amino acid neurotransmitters, including glutamate, GABA and glycine were conserved throughout different phases of the lamprey lifecycle. Functional analysis of glutamate channels with AGB also suggests amino acid signalling is a plesiomorphic feature of the vertebrate retina. Calcium protein cell markers including recoverin, CalB and CalR were highly conserved supporting an essential role for calcium in the retina. On the other hand, increase levels of neurotransmitter precursors, glutamine and aspartate were observed between the two migrant phases suggesting alterations in the *G. australis* metabolic requirements during migration. This was supported by glutamine synthetase labelling, which demonstrated extensive changes in Müller cells between downstream and upstream migrants. Overall, this suggests retinal anatomical changes associated with adaptation of the *G. australis* retina to ocean environments is coupled with changes in metabolic elements including supporting glia and amino acids involved in glutamate production.

## Supporting Information

Figure S1 **Distribution of protein kinase C-α (PKCα) in the retina of** ***G. australis*** **.** PKCα immunoreactivity in the **A:** downstream (DS) migrating and **B:** upstream (US) migrating *G. australis.* Abbreviations are as in Figure 2. Scale bar is 50 µm.
(TIF)
