# Correction: Retinal Amino Acid Neurochemistry of the Southern Hemisphere Lamprey, *Geotria australis*

**DOI:** 10.1371/annotation/99baec7f-908c-4e88-8a87-a25f2aa1630c

**Published:** 2013-10-30

**Authors:** Lisa Nivison-Smith, Shaun P. Collin, Yuan Zhu, Sarah Ready, Monica L. Acosta, David M. Hunt, Ian C. Potter, Michael Kalloniatis

The supporting information file Figure S1 is the wrong file. Please view the correct Figure S1 file here: 
