# Supplementary figures and images for: Retinal Amino Acid Neurochemistry of the Southern Hemisphere Lamprey, Geotria australis
Source: PLoS One. 2013 Mar 13;8(3):e58406. doi: 10.1371/journal.pone.0058406 (PMC3596384; doi:10.1371/journal.pone.0058406)

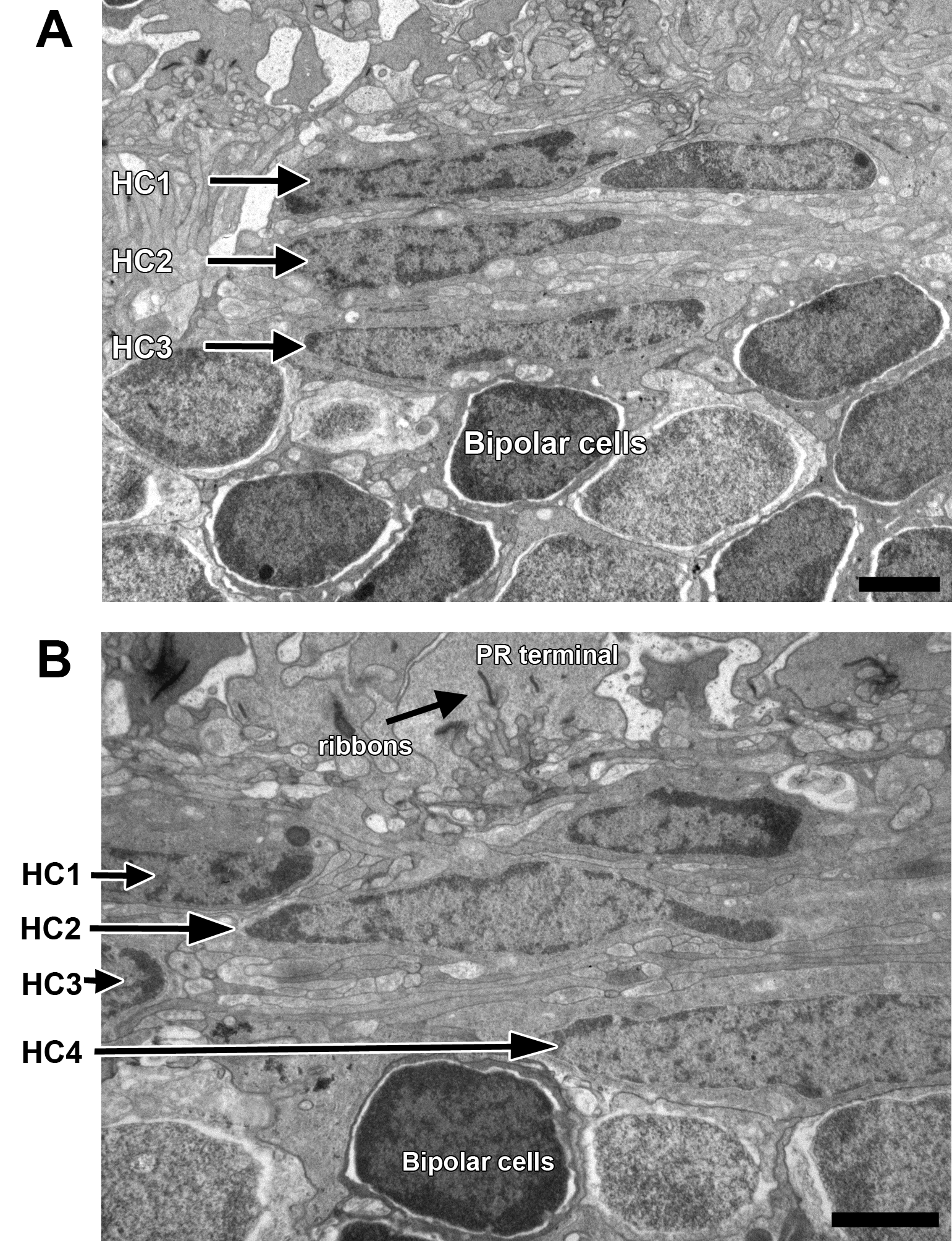

Supplement: Figure S1 — Distribution of protein kinase C-α (PKCα) in the retina of G. australis . PKCα immunoreactivity in the A: downstream (DS) migrating and B: upstream (US) migrating G. australis. Abbreviations are as in Figure 2. Scale bar is 50 µm. (TIF) [file pone.0058406.s001.tif]
