# Supplementary figures and images for: Correction: Retinal Amino Acid Neurochemistry of the Southern Hemisphere Lamprey, Geotria australis
Source: PLoS One. 2013 Oct 30;8(10):10.1371/annotation/99baec7f-908c-4e88-8a87-a25f2aa1630c. doi: 10.1371/annotation/99baec7f-908c-4e88-8a87-a25f2aa1630c (PMC3815031; doi:10.1371/annotation/99baec7f-908c-4e88-8a87-a25f2aa1630c)

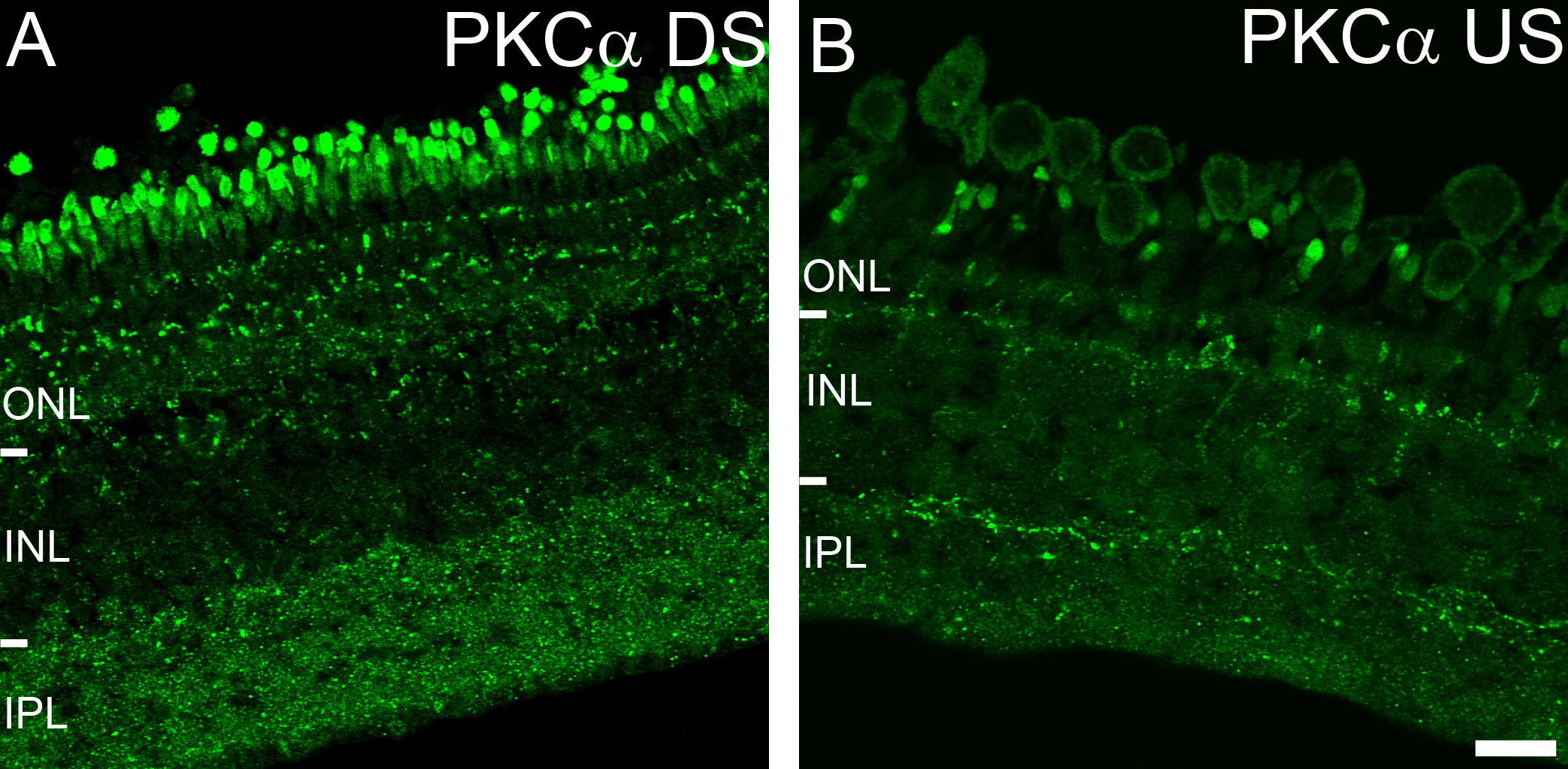

Supplement: Supplementary file 1 [file pone.99baec7f-908c-4e88-8a87-a25f2aa1630c.s001.tif]
